# Cell Immortalization: In Vivo Molecular Bases and In Vitro Techniques for Obtention

**DOI:** 10.3390/biotech12010014

**Published:** 2023-01-28

**Authors:** Javier Curi de Bardet, Celeste Ramírez Cardentey, Belkis López González, Deanira Patrone, Idania Lores Mulet, Dario Siniscalco, María de los Angeles Robinson-Agramonte

**Affiliations:** 1 Department of Neurobiology, International Center for Neurological Restoration, Havana 11300, Cuba; 2 Department of Virology, Tropical Medicine Institute Pedro Kouri, Havana 11400, Cuba; 3 Department of Allergy, Calixto Garcia General University Hospital, Havana 10400, Cuba; 4 Department of Experimental Medicine, Division of Molecular Biology, Biotechnology and Histology, University of Campania, 80138 Naples, Italy; 5 Ramon Gonzalez Coro Hospital, Havana 10400, Cuba; 6 Department of Immunochemical, International Center for Neurological Restoration, Havana 11300, Cuba

**Keywords:** Hayflick limit, telomeres, telomerase, alternative telomere elongation, immortalization

## Abstract

Somatic human cells can divide a finite number of times, a phenomenon known as the Hayflick limit. It is based on the progressive erosion of the telomeric ends each time the cell completes a replicative cycle. Given this problem, researchers need cell lines that do not enter the senescence phase after a certain number of divisions. In this way, more lasting studies can be carried out over time and avoid the tedious work involved in performing cell passes to fresh media. However, some cells have a high replicative potential, such as embryonic stem cells and cancer cells. To accomplish this, these cells express the enzyme telomerase or activate the mechanisms of alternative telomere elongation, which favors the maintenance of the length of their stable telomeres. Researchers have been able to develop cell immortalization technology by studying the cellular and molecular bases of both mechanisms and the genes involved in the control of the cell cycle. Through it, cells with infinite replicative capacity are obtained. To obtain them, viral oncogenes/oncoproteins, myc genes, ectopic expression of telomerase, and the manipulation of genes that regulate the cell cycle, such as p53 and Rb, have been used.

## 1. Introduction

Leonard Hayflick and Paul Moorhead observed in an in vitro study that human somatic cells can carry out a finite number of cell divisions, from which cell cycle arrest is triggered [1]. This study suggested that cells had a mechanism for counting the number of cell divisions, which was not causally related to time but to the number of replicative cycles [2]. However, it was not until 1965 that Hayflick postulated this theory, now known as Hayflick’s limit [3].

The molecular basis on which this phenomenon is based is the progressive shortening of telomeric DNA. Each time the cell completes a replicative cycle, telomeres shorten due to the terminal replication issue. In this way, when the telomere reaches a limited length, the mechanisms of cellular apoptosis and replicative senescence are triggered [4]. However, embryonic stem cells (ESCs) and cancer cells can keep the length of their telomeres stable, evading senescence and apoptosis and acquiring cellular immortality. Two molecular mechanisms associated with the maintenance of telomeric length have been elucidated in early studies: (1) transcriptional activation of the telomerase enzyme and (2) alternative lengthening of telomeres (ALT). The former is found in almost 90% of cancer cells, while the latter is the remaining percent [5,6].

Due to the Hayflick Limit and to obtain more consistent work material over time, researchers need primary cultures with greater replication capacity (immortal cells) that, after several cell divisions, do not enter the senescence phase. The objective of this review is to address the issues associated with those molecular processes that produce the phenomenon of immortalization in vivo and describe the techniques used to obtain them in vitro.

### 1.1. Telomeres

**Figure 1:**
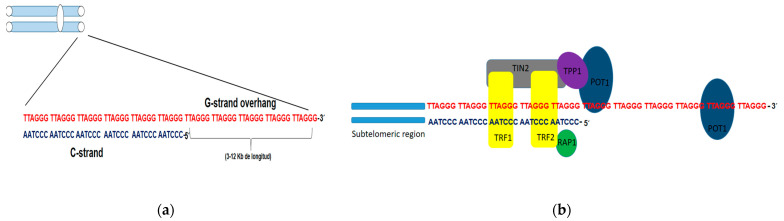
Structure of telomeres in humans. (**a**) Schematic representation of telomeric DNA in humans, composed of tandem repeats of the hexanucleotide sequence TTAGGG (G chain) and its complementary sequence in chain C. The G chain can extend 3–12kb in length (G-strand overhang). (**b**) Adjacent to the telomeres are the sub telomeric regions, also rich in repetitive DNA. Schematic representation of the shelterin or telosome complex. The TRF1 and TRF2 proteins have a binding domain for double-stranded DNA, whereas POT1 can only bind to single-stranded DNA. RAP1 exerts its function on the telomere through a TRF2-binding domain. TIN2 has binding domains for both TRF1 and TRF2, and through another domain, it binds to the TPP1-POT1 complex.

Telomeres are nucleus-protein complexes located at the ends of the chromosomes of eukaryotic cells [7]. This structure prevents the linear ends of chromosomes from being recognized as double-strand DNA breaks(DSBs) [8]; their shortening constitutes a mechanism of tumor suppression by activating cellular senescence signals [9]. In humans, telomeric DNA is composed of tandem repeats of the hexanucleotide 5′-TTAGGG-3′sequence. This sequence extends from 3 to 12 kb in length, giving rise to a single chain rich in guanine residues (G strand) (Figure 1a). The protruding G chain can bend and invade the telomere double chain region and generate a loop structure, the T-loop, thathides the 3′end, as a primitive mechanism for telomere protection [10].

Associated with these tandem repeats is a six-protein complex called shelterins, including TRF1, TRF2, TIN2, RAP1, TPP1, and POT1 (Figure 1b). TRF1 and TRF2 contain a binding domain for dsDNA, while POT1 interacts with telomeric DNA. The other protein complex components do not have binding domains for telomeric DNA and exert their function through interaction with the TRF1 and TRF2 proteins. Shelterins prevent the DNA damage response mechanisms that DSBs activate from being triggered and in this way, prevent the occurrence of homology-directed repair (HDR) or binding processes non-homologous end joining (NHEJ). Likewise, TRF2 protein plays a key role in telomere protection since it makes the construction of the T-loop possible, giving telomeric DNA stability and inaccessibility at its ends. Therefore, telomeres devoid of shelterins lead to the fusion of the ends of chromosomes recognized as DSB that need to be repaired [11,12]. Even though telomeric shortening occurs after each cell cycle, other factors, such as oxidative stress, can influence the speed of telomeric shortening [13,14].

### 1.2. Mechanism Involved in Lengthening of Telomeres

#### Telomerase

**Figure 2:**
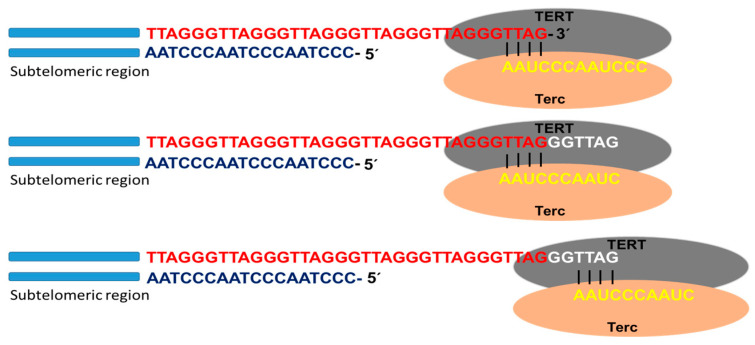
Telomerase activity. Schematic representation of telomere replication by telomerase, as well as the catalytic subunit with reverse transcriptase action (TERT) and the subunit containing the RNA template for telomere replication (Terc). Once the template RNA hybridizes with the telomeric DNA sequence of the G chain, at the 3′-OH end, the telomere polymerization process begins. Once the hexanucleotide sequence is added, the enzyme performs a translocation movement, and telomere elongation continues.

Telomerase is the specialized DNA polymerase responsible for the maintenance and elongation of telomeres. It consists of a ribonucleoprotein with a catalytic subunit with reverse transcriptase action (TERT) and an RNA fragment of sequence complementary to telomeric DNA (Terc: telomerase RNA component). Associated with the complex is dyskerin, a protein that contributes to its formation and stability. The catalytic site of telomerase adds deoxyribonucleotides using the RNA molecule as a template that hybridizes with the telomeric DNA strand (3′-OH end of the G strand). Through this mechanism, the enzyme is able to lengthen the telomere using a translocation movement each time it incorporates the complete hexanucleotide sequence (Figure 2). Once the elongation of the G strand has concluded, the replication of the C strand occurs by the conventional system of polymerases. In this way, the telomeric shortening product of the terminal replication problem is solved [15,16,17].

TERT is highly expressed in cells with high replication and self-renewal capacity, such as ESCs, germline cells, and most cancer cells. However, in adult stem cells (ASCs) and in activated lymphocytes, the activity of this enzyme was also demonstrated, although with much lower expression levels than in embryonic cells [5,18]. Except ASCs and activated lymphocytes, in the other somatic cells, telomerase activity decreases over time after birth, and subsequently, telomeres are shortened with cell divisions [19]. Therefore, telomeric shortening limits the capacity for replicative expansion in telomerase-negative cells, constituting a barrier to achieving cellular immortality and functioning as a biological clock [20]. On the other hand, there is evidence that even in somatic stem cells, telomerase levels are not satisfactory in avoiding telomeric shortening in the course of cellular aging [21].

After 50 or 60 cell cycles, telomeric shortening leads to replicative senescence due to genomic instability associated with fusion events and chromosomal breakage. However, to avoid genetic chaos, some cells can overcome this phase by acquiring mutations in p53 and Rb genes and other genes, encoding for proteins linked to cell cycle control mechanisms and telomerase activation. A small part of the cell population acquires immortality through this pathway and proceeds to carcinogenesis [20,22].

Studies in mice highlighted that the defects in telomeres, and following telomerase re-activation, they are able to trigger the induction of malignant tumors [23]. On the other hand, Barthel and colleagues observed the expression of TERT was present in almost 75% of the analyzed tumor samples, with 31% of samples exhibiting alterations in the TERT promoter and 53% showing methylation patterns [24]. Aberrant expression of TERT is due to mutually exclusive mutations in the TERT promoter (TPM) (−57 A > C; −124 C > T; −138/−139 CC > TT; −146 C > T) that produce transcription factor binding sites of the ETS (E26 transformation-specific) family, such as GA binding protein (GABP) [24,25]. These mutations are mostly heterozygous and induce the allele-specific re-expression of TERT; recruiting GABP promotes an epigenetic change at the chromatin level, passing to its active form [26].

TPMs are the most common non-coding driver of cancer mutations [27]. Indeed, approximately 85% of skin tumors carry them [28]. Likewise, they are coupled with a high expression of TERT and with poorer survival rates in several tumors, such as glioblastoma [29] and meningioma [30]. Furthermore, tumors that carry TPM shave a high risk of recurrence [31]. Similarly, it is worth noting the existence of other pathways able to promote TERT expression during tumor, such as amplifying the TERT gene, hypermethylation of the promoter, and chromosomal rearrangement [24,32].

Moreover, the induction of the molecular mechanisms of DNA repair is also an important factor determining the cellular sequela in response to telomere changes. In this condition, NHEJ activation triggers chromosomal fusion, while the induction of the HDR mechanism could mediate the telomere lengthening through the ALT pathway [20]. Consequently, activation of the telomere repair mechanisms could impact the progression of genomic instability and cancer induction by triggering the end’s union or obtaining cellular immortality through the ALT pathway. Because chromosome fusions and breakage of fused chromosomes could participate in cancer induction, further studies on DNA repair mechanisms that induce chromosomal fusions are required to better understand the role of telomeric shortening in cancer initiation [20,22].

## 2. Alternative Telomere Lengthening (ALT)

Even when telomerase is essential for obtaining cellular immortality, 10–15% of cancer cells can maintain stable telomere length through the ALT pathway [6]. The ALT mechanism is common in sarcomas and tumors of the central and peripheral nervous system but rare in common cancers such as breast cancer, colon cancer, and lung cancer and absent in lymphoma and thymoma [33]. Among cancers that arise in mesenchymal tissues, it was shown that 47% of osteosarcomas and 35% of soft tissue sarcomas (STS) use this mechanism of telomeric lengthening [34]. Furthermore, several differences between and within STS have been described [35]. In liposarcomas (LPS), ALT-positive tumors frequencies from 0% for well-differentiated LPS (characterized by amplification of the 12q13-15) to 80% for pleomorphic LPS, which show elevated levels of complex genomic alterations [36].

Some evidence suggests that the ALT mechanism could be present in normal somatic cells. This was seen early in studies in mice [37] and furthermore in primary human cells where the mechanism based on homologous recombination that resembles ALT seemed to be activated, transiently, in response to oxidative stress [38] or X-ray damage [39]. Similarly, intra-tumor heterogeneity was found in terms of telomere maintenance mechanisms (TMM) in neuroblastomas [40] and osteosarcomas [41], where several telomerase-positive cells have been detected, together with ALT-phenotyped cells.

In pluripotent stem cells (PSCs), telomere maintenance is carried out through telomerase expression, although the ALT pathway was found to play a fundamental role [42,43]. On the other hand, telomeres and the regulation of their length show marked differences between cancer cells and PSCs that use this mechanism. For example, tumor genomes employing the ALT pathway are unstable, exhibiting heterogeneous and dysfunctional telomeres, whereas PSCs possess long telomeres with a stable genome. The underlying mechanisms are not yet clear, but the ALT pathway is activated in PSCs by changes in epigenetic reprogramming [43].

### 2.1. Phenotypic Markers of ALT Cells

Heterogeneous length of telomeres characterized ALT cells in cancers; this length ranges from extremely short (<1 kb) to very long (>20 kb) [44]. In addition, they also show the presence of promyelocytic leukemia bodies (PML) associated with ALT (APBs). These specialized protein bodies contain telomeric DNA and proteins related to molecular recombination events (MRN, WRM, BLM, RAD51, RAD52, among others) [45,46,47]. Another biomarker of these cells are the extrachromosomal telomeric repeats (ECTR) that can be of circular morphology of single (circle C or G) or double (circle T) chain, although they have also been found to be linear in morphology. In the case of the T circle, it can be formed through intra-chromosomal recombination events. In contrast, the mechanism by which circles C or G are formed has not yet been fully elucidated. However, it is believed that circular ECTRs may constitute the substrates for telomeric DNA elongation by the ALT pathway [48]. Finally, ALT activity is associated with a high frequency of telomere-sister chromatid exchanges (T-SCE), which supports the hypothesis that this pathway uses HR as a molecular mechanism to elongate telomeres [49].

### 2.2. Activation of the ALT Pathway

**Figure 3:**
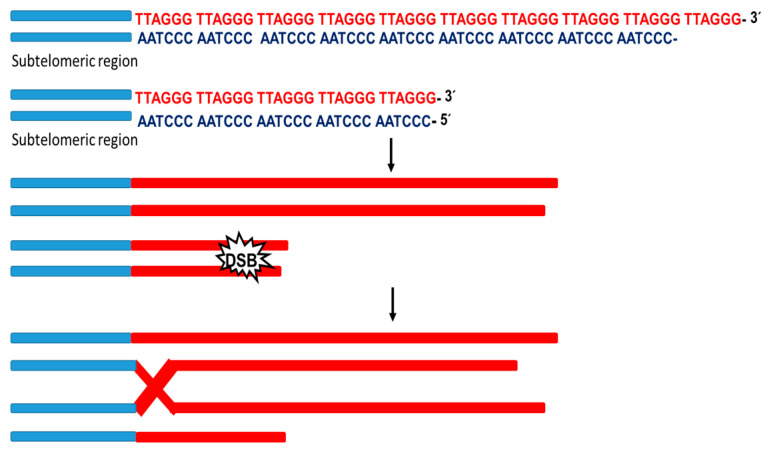
BIR mechanism. Schematic representation of the telomere replication process induced by dsDNA damage. When the dsDNA is damaged, repair mechanisms are activated at the ends of the telomeres. The damaged strand invades the telomeric region of the donor and serves as a primer for the replicative process initiation.

DSBs at the terminals of chromosomes trigger telomeric DNA synthesis through the break-induced replication(BIR)mechanism. BIR is a unique HR pathway able to repair one-ended double-strand breaks (DSBs).Indeed, as a consequence of the broad homology, at the ends of the telomeres when DSB occurs, a BIR mechanism begins where a damaged terminal invades the donor telomere serving as a primer for the initiation of DNA replication [50] (Figure 3). Likewise, it was reported that the replication stress response protein (SMARCAL1) associates with telomeres to block DSB repair and ensure telomere integrity through the ALT pathway. This shows that the resolution of replication stress is a crucial step in this pathway [51]. Furthermore, in the absence of telomerase, telomere shortening leads to the accumulation of ssDNA. The HR machinery can counter this phenomenon and lengthening the telomere through the response pathway for DNA damage [52]. Therefore, telomerase-negative cells require the expression of proteins related to the HR process, indicating that HR is essential for the conservative telomere replication process [53].There are three routes for telomeric elongation by HR: (1) equivalent telomeric exchange between sister chromatids; (2) unequal telomeric exchange between sister chromatids; (3) telomeric exchange between non-sister chromatids. In the case of the second route, it leads to the formation of telomeres of heterogeneous length without gaining in telomere length. However, the third route allows an increase in telomeres’ net length, which is initiated by interchromatin HR and break-induced replication [54].

Two distinct pathways have been identified in ALT cells [55] (Table 1). The first mechanism requires the RAD52 protein, which binds to DNA, promoting the annealing process among complementary DNA strands [56,57]. It was also identified that the RAD52 enrollment to the telomere is a process dependent onSLX4 [58] and that SLX4 is able to process the intermediates of replication formed in the absence of RAD52. If both proteins are simultaneously deleted, mitotic fidelity is not ensured, and dysfunctional telomeres are formed. This phenomenon leads to the accumulation of unresolved stalled forks and the formation of recombination intermediates that can inhibit DNA synthesis and gradually shorten telomeres [56]. The second mechanism is Bloom RecQ DNA helicase (BML)- and POLD3/4-dependent and RAD52-independent, indicating that its activation is under BIR control [50,55]. However, both mechanisms are APBs-mediated, and the repair synthesis pathways depend on the telomere lesions, as well as the phases of the cell cycle [55,56].

The BIR mechanism works through the RFC-PCNA-Polδ axis, independent of other components of the canonical replisome(i.e., ATM, ATR and RAD 51). Furthermore, the BML-TOP3A-RMI complex is mandatory for the synthesis of ALT-mediated telomere. In this mechanism, the intermediates generated by recombination can initiate the large-scale POLD3-dependent telomere synthesis without inducing T-SCE. The SLX4-SLX1-ERC4 complex can inhibit this process, promote the resolution of recombination intermediates, and trigger the telomeric exchange without their extension [59].

## 3. Coexistence of Telomerase and the ALT Pathway

The fusion of cell lines that express one of the two mechanisms allowed for obtaining hybrids where once the genome was stabilized, only one of the mechanisms remained active. Therefore, hybridized cells show only one telomere maintenance mechanism (TMM) by the expression of telomerase or by the activation of the ALT pathway [60,61,62]. However, the ectopic overexpression of telomerase in ALT cells results in obtaining cell lines where both mechanisms coexist. In these cells, telomerase lengthens the shorter telomeres, while APBs and T-SCE can be detected with possible involvement in the maintenance of another subset of telomeres [60,63]. Therefore, the co-existence of both TMMs is only observed in the same cell when telomerase is over-expressed. Meanwhile, it has been shown that they can co-exist within the same tumor in different cells [18]. The coexistence of ALT-positive cells and telomerase has been reported in several types of tumors, which supports the concept of intra-tumor heterogeneity. Indeed, TMM activation is not necessary for initial tumorigenesis. Still, it may occur during tumor expansion, involving the activation of both ALT and telomerase in different types of cells from the same tumor. Conversely, all early-phase tumor cells could have activated one of the two TMMs, with the possibility of changing this choice later [64].

### 3.1. In Vitro Cell Immortalization

When primary cells reach senescence after a few cell divisions, there is a need to reestablish fresh cultures from tissue explants. This task in itself is a tedious process that could also add significant variations from one culture to another. Primary cells should possess extended replicative ability to obtain reliable working samples, such as immortalized cells. Ideally, these cells are capable of prolonged proliferation and show a genotype/phenotype similar or identical to their parent tissue. They play a key role in growth, differentiation, and senescence mechanisms.

**Figure 4:**
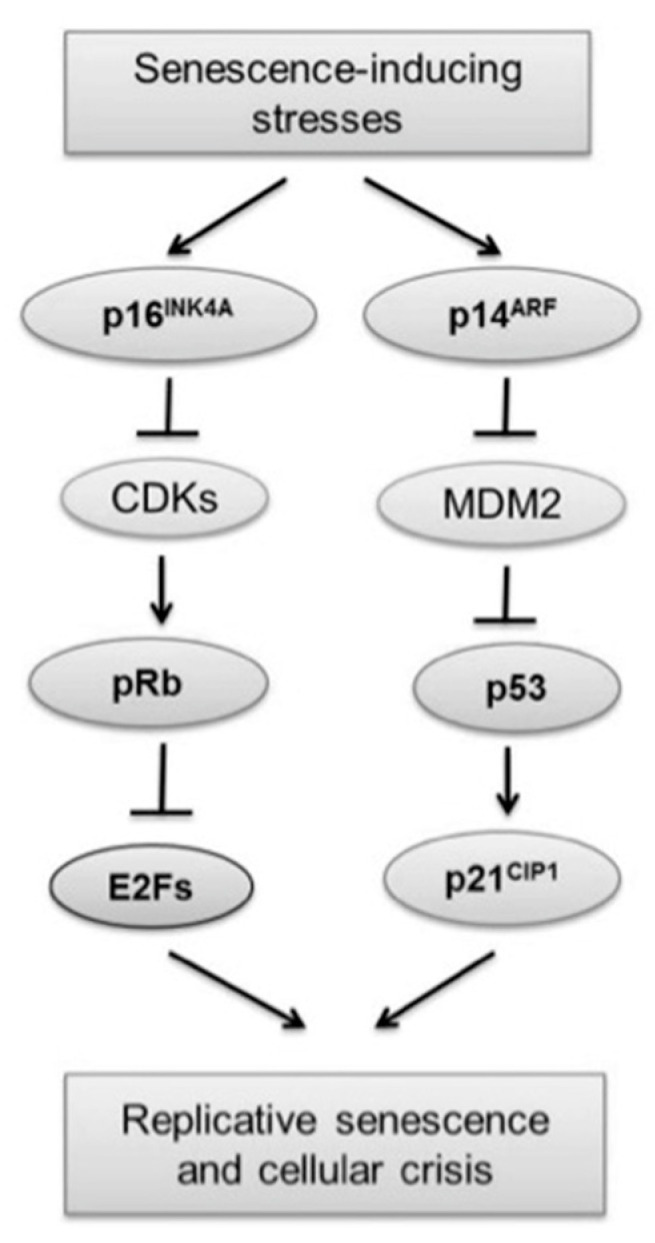
Role of p53 and Rb proteins in senescence and immortalization. Reprinted with modifications from [66] under the terms of the Creative Commons Attribution Non-Commercial License (http://creativecommons.org/licenses/by-nc/4.0, accessed on 25 January 2023).

In obtaining immortalized lines, various biotechnological methods aimed at manipulating the cellular genome have been used. The introduction of viral oncogenes/oncoproteins and the TERT component are the main techniques for such genetic manipulation [65] (Table 2). Immortalization induced by oncogenic viruses is closely linked to the inactivation of proteins that regulate cell cycle progression (p16, p14, p21, p53 and Rb) (Table 2). Through this pathway, viral oncoproteins lead to the inactivation of tumor suppression mechanisms, and they can even activate the expression of telomerase. In addition, a relationship has been observed among pRb inactivation, cellular aneuploidy, and chromosomal instability. Likewise, the signaling pathways activated by several cell strains, in the same way, are channeled onto the pRb and p53 proteins (Figure 4). Hence, pRb and p53 are considered the most important proteins that govern replicative senescence. An active hypo phosphorylated form of pRb is present during senescence, where it binds to members of the E2F family of proteins to inactivate the transcriptional activation of some G1/S phase transition genes. It should be noted that the growth-suppressive activity of Rb apparently remains independent of p53. Then, in human cells, p53 couldinitiatepRb- independent senescence [66].

The p16 protein is a cyclinD/cyclin-dependent kinase (CDK) inhibitor of complexes 4 and 6. It is responsible for initiating an early stage of stress-induced senescence in keratinocytes. In addition, it is one of the most frequently inactivated in human tumors. While p53 and p21 are activated to initiate the senescence response, p16 appears to act to maintain this state. The p16 response was also found to be more exacerbated in human cells than in mouse cells and provides an additional safety barrier to prevent tumor development [66].

In the case of p14, it maintains p53 by sequestering MDM2 (Mouse double homolog minute 2), an E3 ubiquitin ligase, thus avoiding MDM2-mediated targeting of p53 for proteolytic degradation. Fibroblasts from mouse embryos could preferentially depend on the p14/p53 pathway; instead, human keratinocytes use the p16/pRb pathway to enhance the senescence mechanism. Given that, the p16/pRb and p14/p53 pathways need to be intact for oncogene-induced reactions [66].

### 3.2. Techniques for In Vitro Cell Immortalization

The SV40 T antigen is currently used for the transfection of different cells, generating immortal lines through the binding and inactivation of p53 and Rb proteins [67]. It is reported in the literature that in primary human mesothelial cells, but not in primary fibroblasts, SV40 T antigen can activate telomerase [68]. For some primary cell types, overexpression of the SV40 T antigen or hTERT is not enough to achieve efficient immortalization. Conversely, SV40 T antigen and hTERT joint expressions were effective in these cells [69].

Among the cell types immortalized by this mechanism are human proximal epithelial cells, which retain the ability to differentiate [70]. On the other hand, García-Mesa et al. were able to immortalize microglial cells to study the latency and regulation of the Human Immunodeficiency Virus (HIV) in the CNS. They retained most of the phenotypic and functional characteristics of the primary glia cells [71]. In contrast, pre-adipocytes showed aberrant differentiation after immortalization with the SV40 T antigen. Indeed, the SV40 T antigen inhibits pre-adipocytes differentiation in adipocytes due to its ability to block the transcription factor p300/cAMP-response element-binding protein (CBP), which is essential for adipocyte differentiation [72].

The small double-stranded DNA virus HPV is also used to immortalize cell cultures. It can infect the epithelial tissue of mucous and skin. High-risk strains (HPV-8,-16,-18,-31) induce malignant progression of damaged tissues, while low-risk strains(HPV-6,-11)provoke benign lesions. High-risk HPV strains encode E6 and E7oncoproteins, which possess transformative abilities. E6 induces activation of telomerase and accelerates the 26S proteasome-mediated p53 degradation, while the E7 protein can inactivate Rb through the prevention of the binding between the pRb and the transcription factor E2F [65].

Trakarnsanga et al. immortalized early adult erythroblasts using these HPV-16 oncoproteins, generating stable cells and supplying red blood cells. These cells successfully differentiated into functional and mature reticulocytes. Once characterized, no difference was found between these reticulocytes and the adult reticulocytes cultured in vitro, without any aberrant protein expression. The authors suggest there is no risk for clinical use when using viral oncoproteins to produce these cell lines since viral oncoprotein expression is lost before terminal differentiation. Furthermore, the resulting product is enucleated; therefore, tumorigenicity is denied [73].

On the other hand, epithelial cells on the surface of the ovaries have also been immortalized through the viral oncoproteins E6, E7, and SV40 T antigen overexpression [74]. Schutze et al. demonstrated that the immortalization ability of HPV genotypes is inversely linked to the instability of chromosomes. Genotypic variants with decreased immortalization ability in vitro require many genetic mutations of the host cell to facilitate immortalization. This could explain the reported differences in the prevalence of the HPV type in the cervix of uterine tumors and corroborate that the modifications in the host cell genome could affect virus-induced carcinogenesis [75]. Transfection of some cell types with the E6/E7 oncoproteins of HPV generates cell lines that conserve much of the characteristics of the cells of the original tissue.

Another group of genes used in cell immortalization are the myc’s. This family consists of a group of oncogenes: c-myc, N-myc, L-myc, and B-myc. The expression of c-myc is confined to proliferating cells, whereas N-myc and L-myc is related to differentiation. Furthermore, this oncogene can cooperate with certain oncoproteins or with the TERT component of telomerase [65]. Of this family of oncogenes, the c-myc gene is the most studied. About 70% of all tumors are characterized by some c-mycdys regulation, affecting the regulation of the nucleus. The general mechanism of c-mycdys regulation in several tumors indicates that this gene is involved in genome destabilization. Noteworthy, p53 is related to c-myc due to the fact that Myc signaling controls p53-dependent apoptosis and cell immortalization [76]. Although the overexpression of c-myc induces DNA breakage, c-myc induces genomic instability avoiding the pro-apoptotic activity of p53 [77]. In addition to p53, c-myc interacts with other mediators of cell death. Over-activated c-Myc take a role in NF-kB-mediated apoptosis. Hence, the inactivity of NF-kB signaling is a prerequisite for the carcinogenesis induced by Myc [78].

De Filippis et al. developed a method for the immortalization of neural stem cells (NSCs) by transfecting them with a retroviral vector that possessed a mutated variant of c-myc (c-myc T58A). The resulting cells showed levels of improved self-renewal, with a proliferative ability and clonogenic potential superior to the cells of the original tissue and to those immortalized with the wild variant of the oncogene, as well as no sign of malignancy. Likewise, they did not present alterations in their ability to differentiate, giving rise to neurons, astrocytes and, more importantly, a high percentage of oligodendrocytes (23%, undocumented yield up to that time). Hence, this new cell line can be used as an ideal tool for the experimental design and clinical trials aimed at demyelinating diseases [79]. On the other hand, Li et al. also obtained an immortalized line of NSCs by transduction with L-myc. This cell line shows self-renewal and multipotent differentiation in neurons, oligodendrocytes, and astrocytes. There was no in vivo tumorigenicity, and the cells were able to adhere to orthotopic glioma xenografts in deficient mice [80]. One of the reasons for working with the L-myc oncogene to generate immortalized lines for potential clinical use is the low tumorigenicity potential [81]. In contrast, Zhang et al. used the Myc and RAS oncogenes in human fibroblasts. However, both oncogenes could not induce the cells to overcome senescence and cell apoptosis, even after a decrease in p53 levels [82].

The risk of oncogene integration into chromosomes still offers several debates. The use of an oncogenic factor transmissible to the host cells is a safety concern. The transduced oncogene in some cells has undergone additional carcinogenesis modifications. A pathway characterized by less phenotypic/karyotypic changes in respect to the use of SV40 is immortalization through the introduction of hTERT [65].

The ectopic introduction of hTERT and the subsequent activation of telomerase has been shown to have the ability to extend life expectancy and in many cases, to immortalize various cells, i.e., human fibroblasts [83] and retinal pigment epithelial cells [84]. Moreover, the only introduction of hTERT could not be sufficient to immortalize other cell types [85,86]. In the same way, the TERT component has also been used for the immortalization of mesenchymal stem cells (MSCs) since they possess self-renewal and differentiation capacity in different cell lines [87,88,89]. However, in monolayer cell cultures, MSCs and fibroblasts avoid inhibition by cell-to-cell contact, adhere to culture dishes, and aim to grow without limits [65,90,91]. Likewise, spontaneous changes in the expression of c-myc have been reported during the in vitro culture of fibroblasts [92].

Although the use of immortalized cells in in vivo therapies is still a concern, there are some cases in which they have been used successfully to restore organs and tissue. Hung et al. injected umbilical cord blood MSCs in a rat model immortalized by hTERT, into the damaged brain. They successfully and efficiently increased at the injury site for two weeks and did not show tumor production in SCID mice after six months of observation [93].

To avoid the expression of oncogenes, conditional immortalization technology was developed. This technology uses an inducible transgene to produce cells that can be consistently expanded when the transgene is activated. With permanent activation of the transgene, the cells should indefinitely divide. Of course, a transgene that is externally controllable should be preferred. Once the target amount of cellular material is reached, the transgene could be inactivated: the cells will return to a normal post-mitotic state. In this way, the cell formulation is safe for clinical use, avoiding the risks of oncogenic mutations acquired by infinite proliferation [94].

Conditional immortalization is reached by the insertion of a modified gene chemically regulated. Under a determined set of conditions, the transgene is activated; in this way, cells are constantly dividing. Cell division is stopped if the chemical is removed, and cells could potentially possess normal conditions. The development of conditionally immortalized cells as therapeutics is a promising tool for medical research. Preclinical studies using viral oncogenes, myc gene and the catalytic subunit of human telomerase already show encouraging results [94].

The c-Myc ERTAM conditional immortalization tool uses the fusion gene encoding a chimeric protein formed by c-myc, and an N-terminal truncated hormonal binding domain of the G525 mutant murine estrogen receptor that cannot bind 17β-estradiol and estrogen but responds to activation with4-hydroxy tamoxifen (4-OHT), a synthetic estrogen-like agonist [95,96]. Conversely, the wild-type variant, G525 mutant hormone-binding domain, has a 1000-fold lower binding affinity to 4-OHT [96]. Given that in a culture condition with 4-OHT, c-myc is activated, and subsequent cell division proceeds, removing 4-OHT from the culture can signal the cells to revert to a non-activated state and could mature as do normal cells.

For the inactivation ofaSV40 T antigen, a temperature-sensitive mutant of T antigen (SV40 tsA58) was used [97]. This variant possesses the same activity as the wild variant at a permissive temperature (33.5 °C) but is biologically inactivated at temperature of 39 °C [97]. Unlike rodent cells, abrogating the checkpoints in human cells with the SV40 T antigen induces the extension of growth ability beyond normal senescence, and the cells undergo cell death [98,99].

The expression of transgenes could be influenced by different vectors. Unlike lentivirus, adenovirus is not able to integrate transgenes into the host genome and, therefore, may only ensure transient expression [65,100]. Site-specific recombination tools have been studied to acquire a more precise cleavage of oncogenes. As an example, Cre/LoxP technology engineers a transgene flanked by LoxP sites and the transgene is activated when the addition of Cre recombinase. This method is not fully efficient [65,101]. Likewise, a Tet activation/deactivation tool enrolls the tetracycline responsive elements (TRE), which comprise a Tet operator with a minimal promoter. Tetracycline or doxycycline activate the transgene and subsequent cell division [102,103]. As a limitation, filtration includes the continuous expression of the transgene at a low level when the technology is disabled [104].

Recently, a new cell immortalization technique has been proposed as genetic engineering method [105]. This method uses clustered, regularly interspaced, short palindromic repeats (CRISPR)/Cas9 gene editing to produce immortalized cell lines. The strategy is based on reproducing the same genetic changes commonly found in tumors provides in vitro immortalized growth. However, this promising tool is not yet used at alarge scale for the production of commercially cell-immortalized lines.

## 4. Conclusions

Cell immortalization is a useful tool in obtaining cell lines with an indefinite replicative potential as long as the conditions and nutrients necessary for their growth are present. Through this pathway, the cell can exceed the Hayflick limit, evading the processes associated with replicative senescence and subsequent cellular apoptosis. Knowing the molecular bases that support this phenomenon in vivo, which is part of cancer characteristics, has allowed this technique to be used in vitro. In this way, several viral oncogenes/oncoproteins, the TERT component of telomerase, and the expression of genes that control the cell cycle are regulated to obtain conditional immortalization. New molecular techniques able to produce conditionally immortal therapeutic cell lines will possess potential curative or regenerative effects for cellular therapies [94]. In addition, using immortalized cell lines overcomes the ethical issues associated with some types of stem cells. Whereas cell immortalization is a valuable tool to pre-clinically study tumorigenesis pathways [106] and drug screening, as an important limitation to their use in clinical trials, the immortalization procedure raises concerns linked to genetic instability and the production of a tumor phenotype. The conditional procedure, through a molecular on–off the system, could overcome this aspect by removing or permanently silencing the immortalization gene prior to transfer to the patient.

The immortalized NTera2 (NT2) cell line has been extensively studied for brain transplantation. These cells are committed through a neuronal lineage and have been safely used in clinical trials for brain damages [107]. Recently, c-myc lentiviral gene transfection has been used for iPSC-derived hematopoietic progenitor cells to obtain immortalized megakaryocyte cell lines under the control of TET-on system. These obtained cells can release platelets for clinical trials [108].

## Figures and Tables

**Table 1:** Alternative Telomere Lengthening (ALT) mechanisms.

	Mechanisms Enrolled
ALT pathways	SLX4/RAD52
	BML-POLD3/4

**Table 2:** Techniques for establishing in vitro cell immortalization.

Strategy	Method
Expression of the catalytic subunit of telomerase	TERT
Induction of viral oncogenes that inactivate cell cycle proteins (p14, p16, p21, p53, Rb)	-SV40 T antigen -Small dsDNA virus HPV -myc’s oncogenes

## Data Availability

No new data were created or analyzed in this study. Data sharing is not applicable to this article.
